# Feasible Distributed Energy Supply Options for Household Energy Use in China from a Carbon Neutral Perspective

**DOI:** 10.3390/ijerph182412992

**Published:** 2021-12-09

**Authors:** Yingxin Zhang, Sainan Wang, Wei Shao, Junhong Hao

**Affiliations:** 1 School of Economy, Beijing Technology and Business University, Beijing 100048, China; wangsainan@btbu.edu.cn; 2 Institute of Thermal Science and Technology, Shandong University, Jinan 250061, China; shao@sdu.edu.cn; 3 School of Energy Power and Mechanical Engineering, North China Electric Power University, Beijing 102206, China; hjh@ncepu.edu.cn

**Keywords:** household energy consumption, carbon neutrality, distributed energy system, high-efficiency, human development index

## Abstract

This contribution firstly proposed the concept of annual average power generation hours and analyzed per capita energy consumption, carbon emission, and the human development index from a macro perspective. On this basis, we compared the average household electrical energy consumption of urban and rural residents based on the data from CGSS-2015 from a micro perspective. The results show the positive correlation between carbon emissions per capita and the human development index and China’s regional imbalance characteristics between household electricity consumption and renewable energy distribution. Therefore, the distributed energy supply system is proposed as an effective complement to centralized power generation systems and is the key to synergizing human development and carbon emissions in China. Moreover, we analyzed the characteristics of distributed energy supply systems in the context of existing energy supply systems, pointing out the need to fully use solar energy and natural gas. Finally, two types of typical distributed energy supply systems are proposed for satisfying the household energy requirements in remote or rural areas of western and the eastern or coastal areas of China, respectively. Two typical distributed energy systems integrate high-efficiency energy conversion, storage, and transfer devices such as electric heat pumps, photovoltaic thermal, heat and electricity storage, and fuel cells.

## 1. Introduction

### 1.1. Background and Status

To fight against climate warming and environmental pollution, carbon peaking and carbon neutrality have become the main energy development strategies worldwide. At present, in China, various industries such as electric power, transportation, construction, steel, etc., have proposed different routes for achieving carbon peaking and carbon neutrality [1]. The carbon emission of electricity generation based on fossil fuel provides a larger proportion [2]. That is, reducing carbon emissions from electricity production processes is vital to achieving carbon neutrality for China. Recently, the primary solution is the improvement of energy utilization efficiency and increase of renewable energy generation. The rapid development of renewable energy, such as wind power and photovoltaic, has formed an integrated energy system with traditional coal-fired power generation and hydroelectric power generation, turning into China’s main centralized energy supply [3].

For the development of the integrated energy system, many studies have explored carbon peaking and carbon-neutral pathways in energy use from technical, economic, and social perspectives [4,5,6,7]. For example, Liu et al. [8] proposed a differentiated model for evaluating the impact of the emissions trading scheme (ETS) on the development of non-fossil energy sources, and it is concluded that the ETS significantly promotes the development of non-fossil energy sources in China, and the higher the carbon price, the stronger the effect of ETS on promoting the development of non-fossil energy sources. Li et al. [9] developed a collaborative hierarchical framework to coordinate electricity and heat interactions and analyzed the impact of carbon tax, electricity and heat demand responses on the outcome of multi-stakeholder interaction problems. The results show a win-win situation for all participants, with significant reductions in total costs and CO_2_ emissions. Zhao et al. [10] investigated the technical and economic feasibility of New York State’s energy transition goals. They developed an energy conversion optimization framework, pointed out that air-source heat pumps and geothermal technologies will provide 47% and 41% of heat demand, respectively, by 2050. Bao et al. [11] proposed a classification method for renewable energy-led distributed energy supply models. They provided an integrated economic and environmental evaluation model and concluded that the biomass waste-based supply model could achieve “zero” carbon emissions and “zero” energy consumption. In addition, it is advisable to promote waste-based energy utilization and wind and solar energy-based supply modes in new rural and remote areas with abundant resources.

Meanwhile, the distributed energy system has become an attractive alternative technology that has received much attention. Especially on the customer side, distributed energy supply systems can be used to increase the flexibility of the customer side and further improve the consumption and utilization of renewable energy [12,13,14,15,16]. For example, Huo et al. [17] innovatively developed an integrated dynamic simulation model and explored possible emission peaks and peak times by scenario analysis and Monte Carlo simulation methods. The dynamic sensitivity analysis shows that GDP per capita, carbon emission factor, and urban residential floor area play an essential role in driving carbon emissions’ peak and peaking times. Zhang et al. [18] reviewed the distributed generation PV policy changes since 2013. They examined their impact on China’s domestic distributed generation PV market, presented a cost and time breakdown for installing distributed generation PV projects in China, and identified the major barriers to distributed generation PV installation. Zhao et al. [19] calculated the internal rate of return (IRR) and static payback period for some distributed PV systems in five cities with different resource zones in China. The effects of relevant policy variables such as subsidies, benchmark prices, tariffs, and taxes on the economic performance of distributed power systems were discussed. Duan et al. [20] analyzed the influence of solar energy substitution for coal-fired power generation on future greenhouse gas emission trajectories and peak arrival times based on the full-spectrum and life-cycle perspective based on an integrated energy–economic–environment model and a simple climate response model. Moreover, from the application and evaluation perspective of the distributed energy system, Zeng et al. [21] described the current situation of distributed energy development in China. Sameti et al. [22] established the optimal design emissions of the district energy system based on a trade-off between annualized total cost and annual CO_2_ and showed that a district energy system with energy storage provides the best solution to environmental and economic problems. Ren et al. [23] studied the feasibility of distributed energy systems for three typical building clusters in one major city in each of China’s five climate zones. Huang et al. [24] constructed a practical evaluation index system that integrates soft and hard competitiveness and classified distributed energy supply system scenarios according to development characteristics. Yoon et al. [25] artificially assessed the possibility of introducing cogeneration distributed energy systems in existing multi-family dwellings. The annual energy consumption of a typical urban multifamily dwelling was estimated based on primary energy consumption reduction, CO_2_ emission reduction, simple payback period, and recurring cost values.

The above existing research on distributed energy supply systems has been carried out from various aspects such as system energy source, process construction, operation strategy, economic evaluation, and application scenarios. Combined with carbon emission requirements, different distributed energy supply schemes are given from cities, industrial estates, buildings, etc. [26,27,28]. These studies provide the necessary basis for the development of distributed energy supply systems. Besides, household energy consumption playing a vital role as main energy consumption on the user side is also significant for carbon peaking and carbon neutrality [29,30,31,32]. For example, Wu et al. [33] provided a systematical overview of rural household energy consumption in China from 1985 to 2013 and illustrated the pattern of rural household energy consumption using a comprehensive household survey, the Chinese Residential Energy Consumption Survey (CRECS, 2013). Zou et al. [34] presented a detailed analysis of rural household energy consumption characteristics based on the data of 1472 rural households from the Chinese General Social Survey of 2015 (CGSS2015). Ren et al. [35] predicted energy consumption and carbon emissions using both the carbon emissions coefficient and the sector energy consumption method and concluded that urbanization positively affects energy consumption and carbon emissions in China. Chen et al. [36] proposed a spatial downscaling framework to identify different provinces and sectors’ roles in promoting carbon emission peaks. Zhang et al. [37] developed a questionnaire survey to investigate and evaluate the carbon emissions of household energy consumption and analyzed the influence factors, including residential consumption, housing conditions, daily travel distance, etc.

### 1.2. Research Gap

Currently, many studies have been conducted on various aspects of planning, modeling, operation, and evaluation of integrated energy system, local energy system, and community energy system by combining with the renewable energy. Meanwhile, some studies have focused more on household energy consumption in rural or urban areas of a particular Chinese province. These researches provide some feasible and significant technological guides for the future energy supply systems from the city-level, community-level, and household-level. However, due to the considerable difference in household energy consumption in each province of China, more research about the choice of household energy supply pathway is desirable and should be developed by simultaneously considering the energy resource distribution and social development characteristics in the context of carbon neutrality. That is, the synergy between household energy consumption and renewable energy distribution in different regions of China is crucial for the current development of distributed energy supply systems and the choice of household energy supply pathways under the carbon neutrality target.

### 1.3. Resrarch Content and Novelty

This contribution analyzes the energy consumption, power generation, carbon emission, and human development index of China near 40 years. It presents the average household electrical energy consumption of urban and rural residents in 28 provinces based on the data from the Chinese General Social Survey 2015 (CGSS-2015). Based on the above macro and micro statistical analysis, we analyze the characteristic of the distributed energy system and present the future energy system that should be the solar energy-based energy supply system. Moreover, two types of typical distributed energy supply systems are proposed by integrating high-efficiency energy conversion, storage, and exchange devices that are feasible pathways for the choice of household energy supply in China from a carbon neutral perspective. The following provides the novelty of the work,

(1) The research proposes a new concept of annual average power generation hours to analyze the development of the energy generation, analyzes the relationship between carbon emission and the human development index in China.
(2) The research first presents the household electricity consumption distribution of urban and rural areas in each province of China based on the CGSS-2015, and obtain the imbalance characteristic between the renewable energy distribution and the household electricity consumption.
(3) The research concludes the characteristics of solar energy-based distributed energy supply system based on the macro and micro analysis, and proposes two types of distributed energy system by integrating clean, high-efficiency, and low/zero carbon technologies for eastern and western areas in China under the carbon neutral perspective.

## 2. Energy Consumption and Carbon Emission in China

### 2.1. Energy Generation and Consumption in China

**Figure 1:**
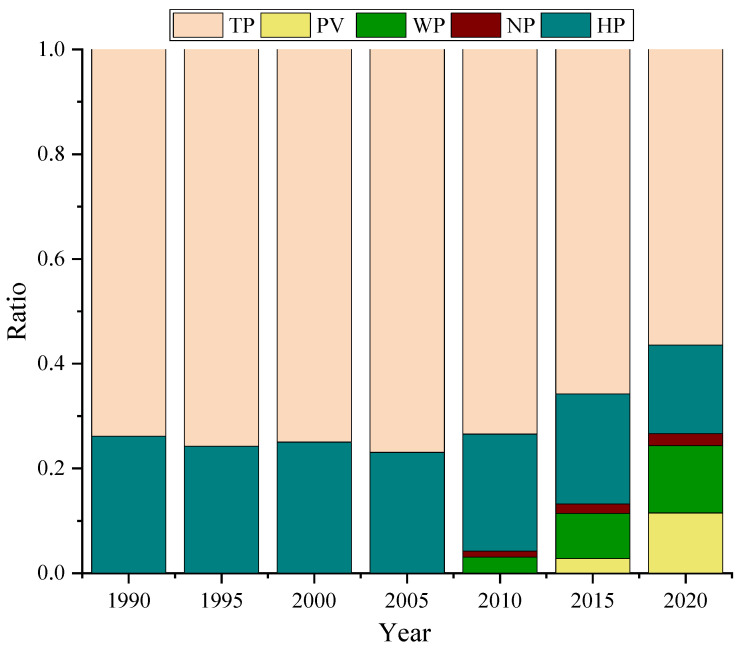
The power generation installed capacity ratio (1990–2020) in China.

In China, thermal power (TP), hydropower (HP), nuclear power (NP), wind power (WP), and photovoltaic (PV) have been the primary generation. Figure 1 shows the variation in the installed capacity share of different power generation methods. The installed capacity of thermal power generation occupies the largest share and has decreased from 73.8% to 56.4% in the last 30 years. The total installed capacity of wind power and PV has increased rapidly in the last decade and has already surpassed the installed capacity of hydropower, reaching 43.2% of the total installed capacity of thermal power in 2020. Currently, coal-fired power generation is still the primary method of electricity production in China. However, last year, for the first time, the installed capacity of renewable energy surpassed that of coal-fired units. Besides, the continuous increase of installed capacity of wind power and PV will become an important step to achieving the carbon peak and carbon neutral strategy in the future.

**Figure 2:**
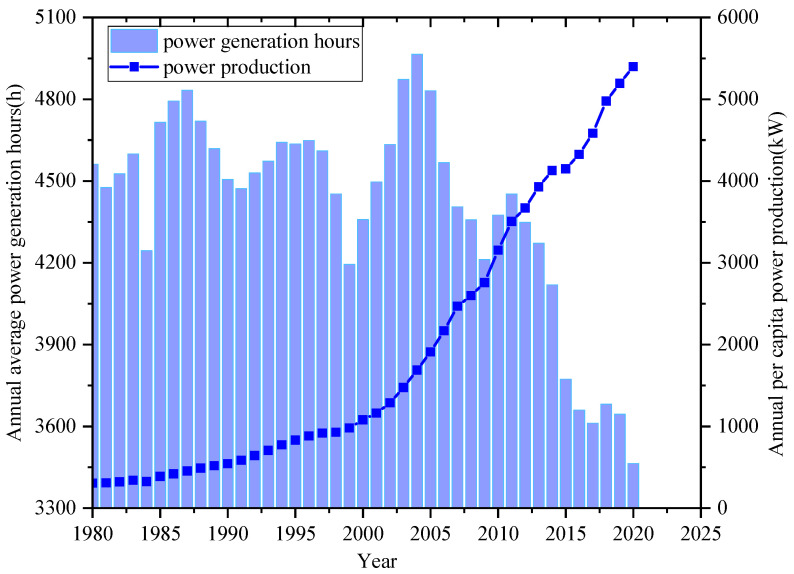
Annual average power generation hours and power production (1980–2020) in China.

**Figure 3:**
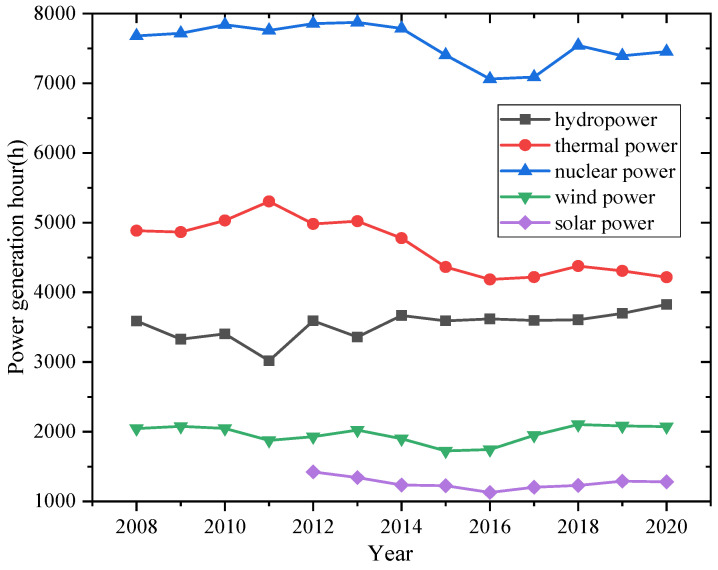
Power generation utilization hours of different power generation ways in China (2008–2020).

Changes in the structure of the installed power capacity of energy will cause changes in the production and consumption of electricity. Figure 2 presents the annual average power generation hours, including all-power generation ways as thermal power, wind power, hydropower, nuclear, and PV shown in Figure 1, and meanwhile shows the annual per capita power production from 1980 to 2020 in China. Figure 3 shows the power generation utilization hours of different power generation ways in China. The annual average power generation hours are a new concept proposed in this paper and equals the total power generation ratio to the total installed capacity. Although the installed capacity of power generation continuously increases, the power generation hours reached up to the maximum 4964.7 h in 2004 and the minimum 3463.6 h in 2020.

On the one hand, the wind power and PV generation installation ratios increase with lower generation hours due to their essential characteristics. On the other hand, the ratio of thermal power generation and the generating equipment availability hour decrease. On the other hand, since 2010, the average annual number of hours of electricity generated has decreased to 91 h per year. The thermal plant has the same trend shown in Figure 3. Especially in 2020, the annual average power generation hours will reduce 180.8 h due to the impact of the COVID-19 pandemic. Besides, the annual per capita power production has continued to grow, increasing nearly 18-fold in 40 years. In particular, it has increased from 3153 kW to 5397 kW in the last decade, almost 71.2%.

### 2.2. Carbon Emission and Human Development Index

**Figure 4:**
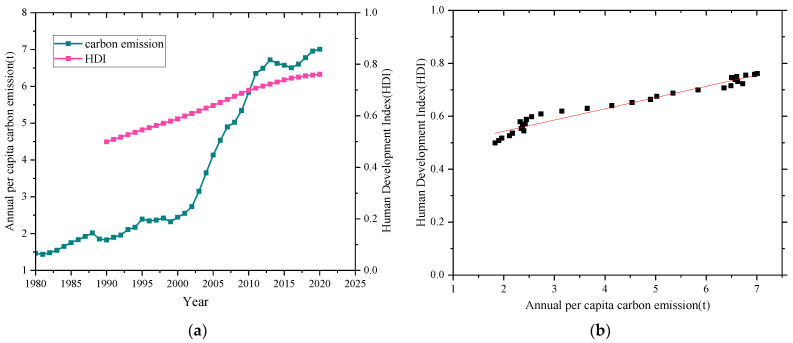
(**a**) Annual per capita carbon emission and HDI of China, (**b**) HDI varies with the annual per capita carbon emission.

In the past four decades, the rapid development of energy and electricity in China has dramatically improved people’s social quality of life, bringing about serious environmental problems. Meanwhile, HDI is an indicator of economic and social development and is a comprehensive evaluation containing the financial standard, education measure, and life expectation [38]. Therefore, Figure 4a presents the annual per capita carbon emission of China from 1980 to 2020 and the human development index (HDI) of China from 1990 to 2020 and their relationship. Over the past four decades, the annual per capita carbon emissions have increased from 1.467 tons to 7.008 tons, an average increase of 0.138 t per year. Besides, the HDI increases from 0.499 to 0.761 from 1990 to 2020 in China. Figure 4b also shows that the HDI is positively correlated with carbon emissions per capita. In other words, to a certain extent, the increase in carbon emissions per capita contributes to the improvement of HDI.

## 3. Household Electricity Consumption Distribution

### 3.1. Household Electricity Consumption in Urban and Rural Areas

**Figure 5:**
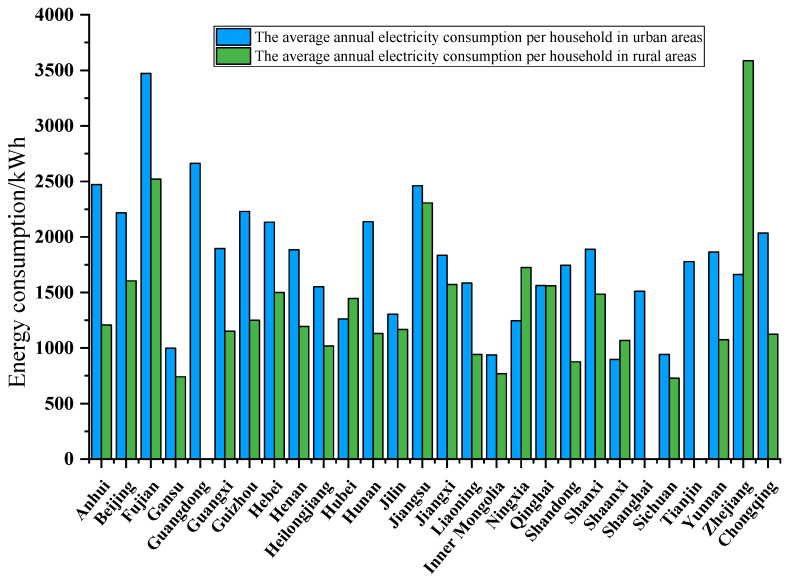
The average annual electricity consumption per household in urban and rural residents in 28 provinces of China, 2014.

The above analysis provides a macroscopic view of China’s energy production and social development from different data such as installed capacity, per capita electricity generation, per capita carbon emissions and human development index. Besides, from the microscopic view, household electricity consumption also greatly influences the total energy consumption in China, in particular, electric power consumption. This article analyzes the corresponding electricity power consumption data based on the 2015 Chinese General Social Survey (CGSS-2015). Figure 5 presents the average household electricity consumption of urban and rural residents in 28 provinces of China, respectively. We can find that the urban electricity consumption is almost more than the rural electricity consumption. According to the statistical data, the average annual electricity consumption per household of urban residents in Fujian Province is the highest. The average annual electricity consumption per household of urban residents in Gansu, Inner Mongolia, Shaanxi, and Sichuan is the lowest in addition to the national position. Geographically, it can be seen that the average annual electricity consumption per household of urban residents in the northern region is slightly lower than that in the south, and the trend of electricity consumption increases gradually from the northwest to the southeast. In addition, the average annual electricity consumption per household of urban residents has a regional concentration distribution pattern, with high electricity consumption areas concentrated in the capital, east China, south China, and southeast coastal areas, which is closely related to the high level of political and economic development in these areas. Meanwhile, considering that the overall development level of the central and northern regions still has more room for improvement, the lower overall electricity consumption in this region also supports this status quo.

**Figure 6:**
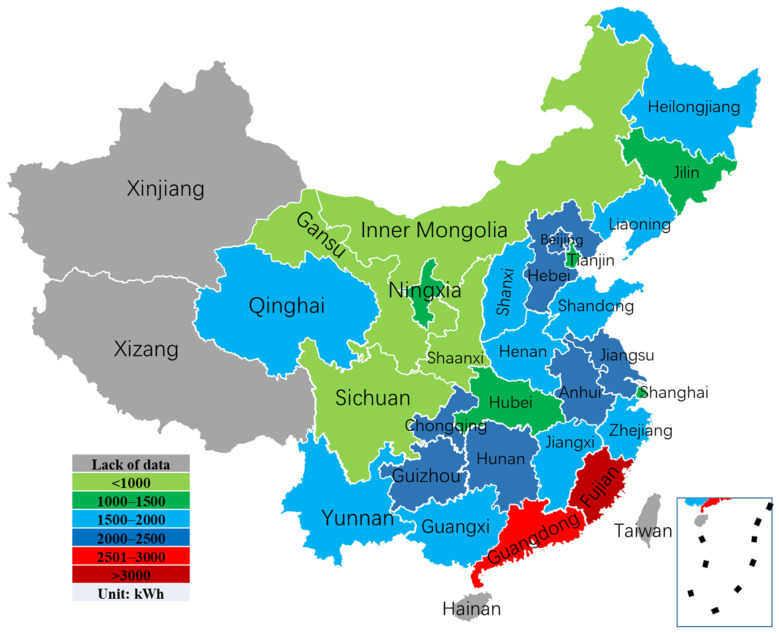
The average household electricity consumption of urban residents in 28 provinces of China, 2014.

Moreover, Figure 6 presents the average household electricity consumption of urban residents in 28 provinces of China, 2014. For urban residents, the average annual electricity consumption per household in Fujian Province, Guangdong Province, and Anhui Province resides in the top three statistical provinces in order. The average annual electricity consumption per household is concentrated in the range of 1500–2000 kWh in more provinces, with 12, which better reflects the average level of urban residents nationwide. For the electricity consumption of rural residents, there are missing data for Guangdong Province, Tianjin, and Shanghai. The average annual electricity consumption per household in Zhejiang, Fujian, and Jiangsu provinces is in the top three statistical provinces in order of residence. Comparing the average annual electricity consumption per household in different regions, we can see that the overall electricity consumption of urban residents is higher than that of rural residents. The average annual electricity consumption per household of urban residents in Shandong Province, Guizhou Province, Hunan Province, and Chongqing City is already about twice that of rural residents, indicating a large imbalance between urban and rural development in the regions as mentioned above. In comparison, the average annual electricity consumption per household of rural residents in Zhejiang Province, Ningxia Province, Hubei Province, and Shaanxi Province is higher than rural residents. The average annual electricity consumption per household in Zhejiang Province, Ningxia Province, Hubei Province, and Shaanxi Province is higher than that of urban residents, which indicates to a certain extent that the development level of local villages and cities is more average and the electricity consumption is comparable. In particular, the average annual electricity consumption per household in Zhejiang Province is more than twice that of urban residents, which laterally indicates that the development level of local rural areas is higher.

### 3.2. Wind and Solar Distribution in China

**Figure 7:**
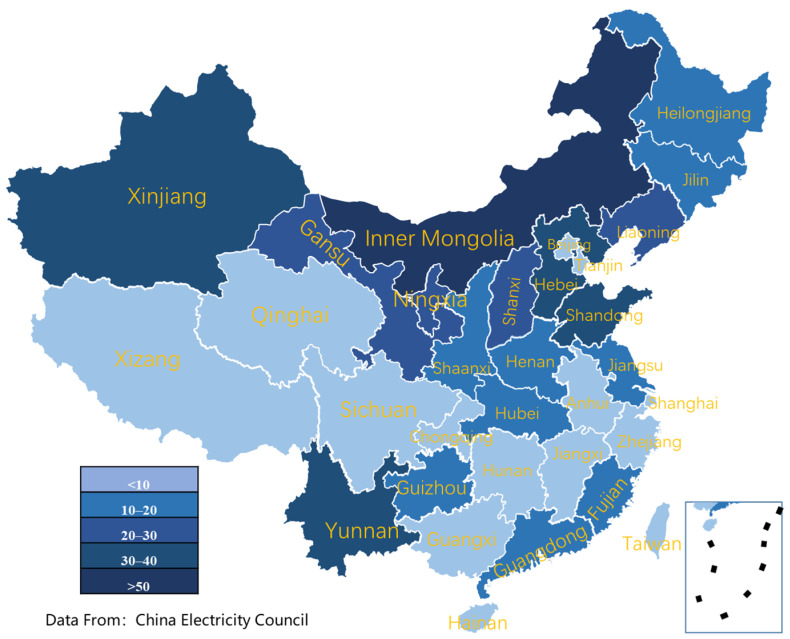
Superior wind power plant distribution in China until 2020.

**Figure 8:**
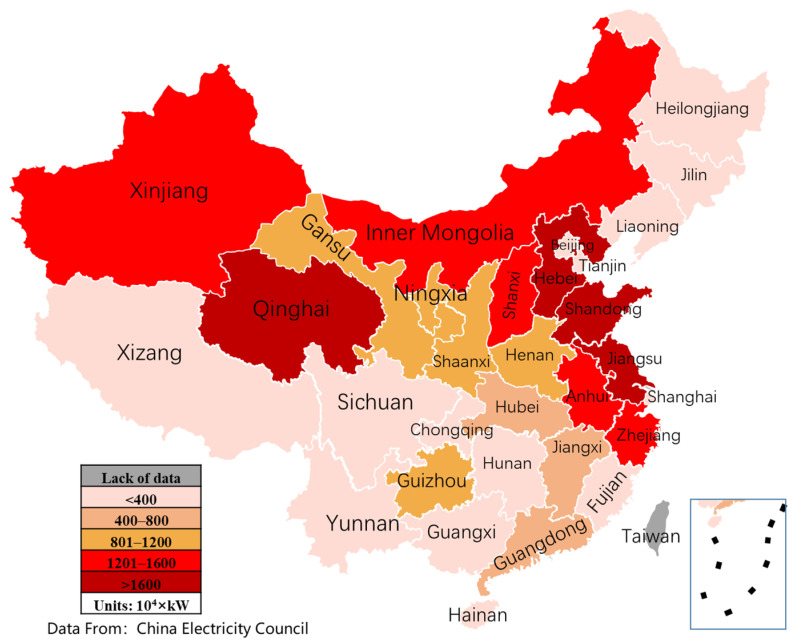
PV installed capacity distribution in China until 2020.

Finally, the more economically developed coastal areas, such as Beijing, Shanghai, Jiangsu, Zhejiang, and Guangdong, have greater household electrical energy consumption on average than the lower Midwest regions in China. Figure 7 and Figure 8 show wind power and solar energy installed capacity until 2020 in China. In addition, more renewable energy sources such as wind energy and solar energy are located in the Midwest of China, such as Xinjiang, Qinghai, Gansu, Inner Mongolia, etc. Therefore, the non-balance between energy generation and energy consumption generates some obstacles for achieving the carbon peak and carbon neutral strategies of China. Therefore, developing an extra-high voltage power grid is an effective solution for improving renewable energy utilization and reducing carbon emissions. Besides, in these energy consumption regions such as Beijing, Shanghai, and Guangdong, distributed energy systems have also become a feasible solution for ensuring energy demands with low or zero carbon emissions.

In summary, from a macro perspective, the installed capacity of power generation based on fossil fuels and renewable energy sources has both continued to increase in the last decade. Although the average annual power generation utilization hours decrease year by year, it is conducive to improving China’s human development index. However, a continuous increase in carbon emissions per capita comes with this. Meanwhile, from a micro perspective, the statistical and comparative analysis of the average household electricity consumption and wind power generation in each province in China shows regional and urban–rural development imbalances in China. There is also a large regional inconsistency between energy production and consumption. Therefore, under the carbon neutral perspective, the selection of future household energy supply paths should fully consider the characteristics of household electricity consumption in different regions, the characteristics of renewable energy distribution, and China’s HDI targets.

## 4. Characteristics of the Distributed Energy Supply System

### 4.1. An Alternative Future Energy System

According to the above macro and micro energy consumption and carbon emission analysis, it can be seen that carbon emission, electricity consumption, and human development index are positively correlated, while there are regional imbalance characteristics of energy consumption and production. Therefore, the development and selection of future energy systems must simultaneously consider human development, regional energy resources distribution, users’ energy demand, etc. The improvement of the contradiction between carbon emission and human development is more significant for China.

**Figure 9:**
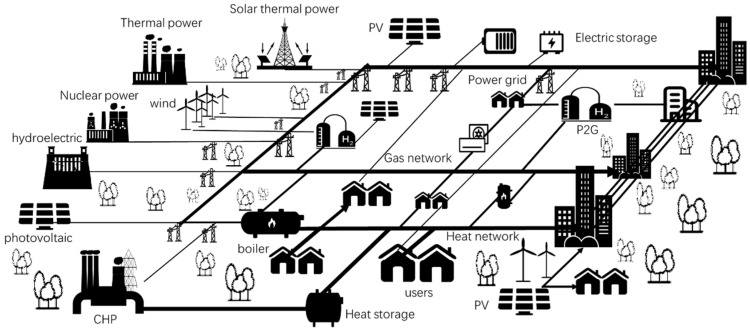
The sketch of the future integrated energy system.

Recently, the energy demands of the industry, commerce, and residential life have focused more on electricity and heat/cool. Various energy generation units provide electricity, heat, and cool. Based on the current feature of China’s energy structure and future development plan, this research provides an alternative sketch of the future integrated energy system under the carbon neutral perspective shown in Figure 9. On the energy generation side, various thermal power, combined heat, and power (CHP), wind power, hydroelectric power, nuclear power, photovoltaic (PV), and solar thermal power are the main power generation ways in China, which can meet large amounts of electricity demand. There are power grid, gas network, and heat network in the energy transport processes. There are electricity, heat, and cool demands on the energy consumption side. Meanwhile, various energy storage units (such as electrochemical energy storage, mechanical energy storage, thermal energy storage, etc.), power to gas units, and electricity to heat conversion units (such as heat pump, electrical heating) are in the integrated energy system. Besides, the power generation units such as wind power and PV are also arranged on the customer side. There are two main types of energy production: centralized and distributed in the future integrated energy system. Meanwhile, the future integrated energy system can achieve efficient, flexible, safe, and stable operation by coordinating source, network, load, and storage.

Based on the data shown in Figure 4, China’s human development needs to be further enhanced, yet the traditional centralized, fossil fuel-based approach to energy utilization needs to change under the strategy of carbon peaking and carbon neutrality in China. In the context of synergistic consideration of China’s social development and carbon neutrality, an energy supply system based on renewable energy is necessary and significant. However, the imbalance characteristic between the renewable energy source and the user demands becomes the main obstacle of renewable energy utilization. Therefore, as a complement to the traditional centralized energy production methods, the development of distributed energy systems is essential to balance the regional contradictions in energy production and consumption and increase the efficient utilization of renewable energy [21,39]. Typically, distributed energy systems are placed on the customer side, and there are various types [40,41,42,43,44]. Therefore, the choice and construct of the distributed energy system are significant and necessary for the household energy consumption in a carbon neutral perspective for different regions in China.

### 4.2. Solar Energy-Based Energy Supply System

It is well known that solar energy is the most widely distributed energy. Therefore, the solar energy-based distributed energy supply system is desirable and will be a significant viable energy supply option for future household energy demand. However, due to its intermittent nature and unavailability at night, the comprehensive utilization of solar energy is provided and required to satisfy the electricity, heat/cool, and gas requirements of users. This utilization can achieve high efficiency, flexibility, low carbon, and security of the distributed energy system.

**Figure 10:**
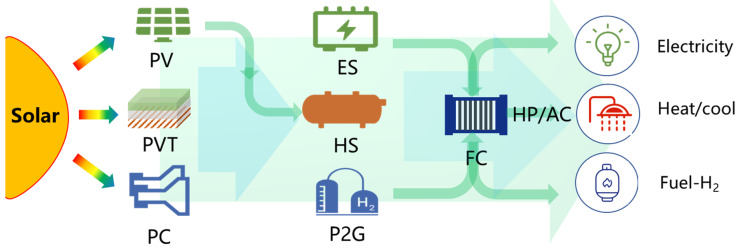
Comprehensive utilization ways of solar energy.

Figure 10 shows the comprehensive utilization ways of solar energy. All energy comes from the sun, and solar energy is capable of producing heat and electricity through photovoltaics (PV) and photovoltaic thermal (PVT), and fuels, such as hydrogen and methanol, through photochemistry (PC) and other technologies. In order to solve the problem of indirectness and uncertainty of solar energy, energy storage can be used through electricity storage (ES), heat storage (HS), power to gas (P2G), and other energy storage methods. Finally, it further meets the different needs of users for electricity, heat, cold, and gas through high-efficient energy conversions such as fuel cells (FC), heat pumps (HP), and air conditioners (AC). As the research from Frankea et al. [45], the interdependencies of renewable energy and flexibility options such as hydrogen and batteries may be the most cost-effective solutions for the future Chinese energy system under different scenarios. China’s carbon-neutral energy system is mainly based on solar power plants that use batteries and hydrogen reconversion to meet nighttime demand.

## 5. Two Types of Distributed Energy Systems

According to the above-mentioned distributed energy system based on solar energy utilization, photovoltaic and photovoltaic thermal utilization can generate different grades of energy such as electricity and heat to meet various needs of users. Whether in urban or rural areas, in western or eastern China regions, solar energy through building walls, house roofs, etc., is a viable way to produce energy. Therefore, the following will propose two types of typical distributed energy supply systems based on wind, photovoltaic and photovoltaic thermal utilization.

### 5.1. Heat Pump and PVT Integration Solutions

**Figure 11:**
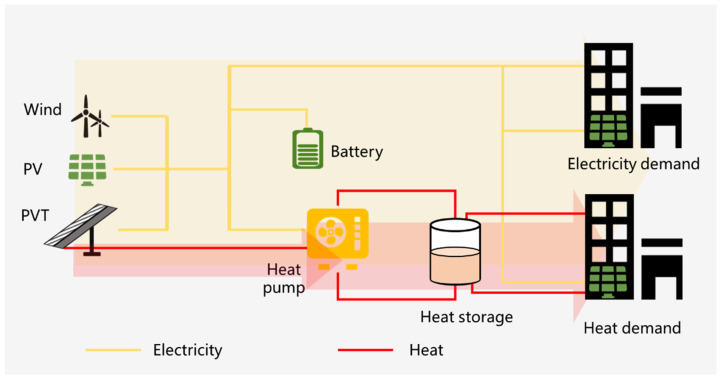
The integration of heat pump, wind, PV, PVT, and energy storage units.

In remote or rural areas of western China, good wind and solar resources are shown in Figure 7 and Figure 8. Meanwhile, the energy and electricity consumption are relatively decentralized, and the demand for electricity and heat from retail households is prominent. Therefore, to satisfy the electricity and heat demands of households, wind turbines, PV and PVT can be integrated to form a distributed energy system with the complementary scenery. As shown in Figure 11, this distributed integrated energy system includes wind turbine power generation, photovoltaic power generation, a high-efficiency electric heating device composed of PVT and heat pump, and energy storage devices such as batteries and heat storage, which together meet the electricity and heat needs of users. For the PVT-heat pump subsystem, the working medium flows through the photovoltaic panels, which on the one hand, reduces the temperature of the photovoltaic back and improves the efficiency of photovoltaic power generation. On the other hand, the heated air goes into the evaporator of the heat pump to further enhance the electric–heat conversion coefficient of the heat pump.

Meanwhile, energy storage is significant for this distributed energy system, including electricity and heat storage devices. The electricity storage equipment can be electrochemical batteries to improve user-side wind power and photovoltaic power generation. In addition, the thermal storage equipment can be water storage or ice storage to meet the different thermal needs of users. That is, this electricity and heat storage device ensures the stable electricity and heat output of this distributed energy system and guarantees the energy demand of customers without carbon emissions.

### 5.2. Fuel Cells and PVT Integration Solutions

Unlike the western areas in China, wind power resources are relatively scarce in the eastern or coastal areas, as shown in Figure 7. Therefore, solar energy should be better utilized in household energy consumption. Due to the intermittent nature of solar energy utilization, a certain amount of energy storage technology is required to guarantee the stability and reliability of energy consumption by users. In addition, solar energy should be stored directly as electricity or converted into fuels, such as hydrogen and methane.

**Figure 12:**
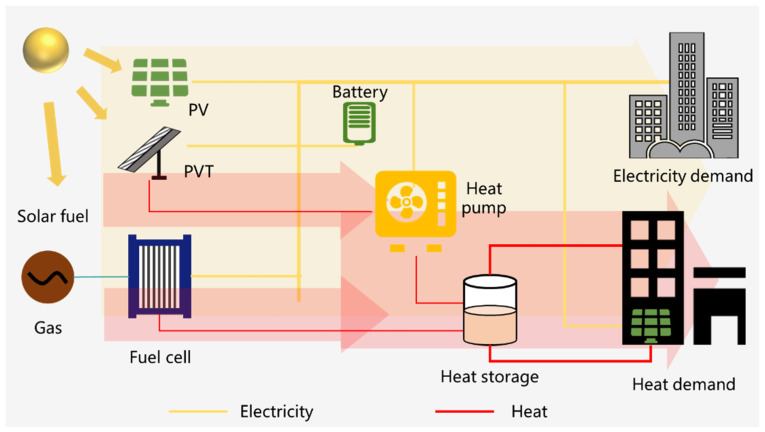
The integration of PV, PVT, FC, and energy storage units.

Therefore, this research proposes an alternative distributed energy system containing PV, PVT, and fuel cells shown in Figure 12. The fuel cell is a high-efficiency combined heat and power generation technology [46,47]. Among them, building PV and PVT can be used to generate electricity. Using natural gas and solar fuel, etc., the combined heat and power supply is carried out by means of fuel cells. For example, fuel cells can use solar fuel (natural gas or hydrogen) to directly convert the chemical energy of the fuel into electricity without producing mission pollutants and can provide both electricity and heat with an efficiency of over 80%. The combination of PVT and heat pump can improve the coefficient of performance of the heat pump that produces the heat for users. Besides, batteries and heat storage are introduced to overcome the temporal mismatches between the solar energy distribution and the electricity and heat needs of the users.

Figure 11 and Figure 12 propose two types of distributed energy supply systems based on wind power and solar power, so they do not have carbon emissions. Meanwhile, these two types of distributed energy supply systems are two possible conceptual solutions based on the basic situation of renewable energy distribution and household energy consumption in China and are not real systems. However, they provide a feasible reference for the design of real systems in the future. In the future, we still need to plan and design, working conditions optimization and operational control from the technical and economic point of view for the actual user needs and the geographical characteristics of the location.

## 6. Conclusions

In the context of carbon neutrality, making full use of renewable energy is key to further improving China’s human development index. To improve China’s HDI under the carbon neutrality constraint, effectively reducing household energy consumption and selecting appropriate household energy supply options are significant. Therefore, this research obtained some conclusions

(1) This contribution analyzed the proportion of installed capacity of thermal, hydropower, nuclear, wind, and photovoltaic power, and firstly proposed and calculated the annual average power generation utilization, annual per capita power generation, carbon emission, and human development index in China in the past four decades, and concluded the positive correlation between energy consumption, carbon emission and social development from a macro perspective.
(2) Based on the 2015 Chinese General Social Survey (CGSS-2015) data, we analyzed the average electrical energy consumption of urban and rural households in 28 provinces across China from a micro perspective. The results show regional imbalance characteristics between household electricity consumption and renewable energy distribution characteristics in China.
(3) In order to better consume renewable energy and promote the carbon neutral strategy, this paper proposes that distributed energy supply system is a feasible option as an effective complement to the centralized power generation system based on the distribution of household energy consumption and scenic resources in the mid-western and eastern regions.
(4) Two types of typical distributed energy supply systems can be provided by integrating efficient energy conversion, storage, and exchange devices, such as electric heat pumps, PV, PVT, heat storage, electricity storage, and fuel cells, all of which are clean, efficient, low-carbon, and safe. In conclusion, the proposed two distributed energy systems can achieve carbon neutrality while meeting the energy needs of households.

## Data Availability

Restrictions apply to the availability of these data. Data was obtained from Chinese General Social Survey and are available at http://cgss.ruc.edu.cn/ with the permission of Chinese General Social Survey.
